# Understanding Police Performance Under Stress: Insights From the Biopsychosocial Model of Challenge and Threat

**DOI:** 10.3389/fpsyg.2019.01800

**Published:** 2019-08-09

**Authors:** Donovan C. Kelley, Erika Siegel, Jolie B. Wormwood

**Affiliations:** ^1^Department of Psychology, University of New Hampshire, Durham, NH, United States; ^2^Department of Psychiatry, University of California, San Francisco, San Francisco, CA, United States

**Keywords:** challenge, threat, biopsychosocial, police, shootings

## Abstract

We examine when and how police officers may avoid costly errors under stress by leveraging theoretical and empirical work on the biopsychosocial (BPS) model of challenge and threat. According to the BPS model, in motivated performance contexts (e.g., test taking, athletics), the evaluation of situational and task demands in relation to one’s perceived resources available to cope with those demands engenders distinct patterns of peripheral physiological responding. Individuals experience more challenge-like states in which blood circulates more efficiently in the periphery when they evaluate their coping resources as meeting or exceeding the task demands. Conversely, individuals experience more threat-like states in which blood circulates less efficiently in the periphery when they view the situation or task demands as exceeding their coping resources. Patterns of response consistent with challenge and threat states have been shown to predict important performance and decision-making outcomes in stressful contexts, and repeated experiences of threat-like patterns of physiological activity are thought to have detrimental effects on long-term cardiovascular health. To date, however, research has not used the biopsychosocial model to understand police decision-making under stress. Here, we review relevant empirical work from the perspective of the BPS model concerning how minority status and power can shape challenge and threat responding and contribute to decision-making under stress. We then detail a research agenda aimed at improving the translational value of research being conducted within the BPS model for understanding complex performance and decision-making in the real world, including among law enforcement personnel.

Police work is often stressful and requires execution of difficult tasks where outcomes are of high consequence. In this paper, we propose that many situations involving police decision-making or performance can be understood as motivated performance contexts: the situations are goal-relevant, involve instrumental cognitive processing, have uncertain outcomes, and require active rather than passive responding (Blascovich and Mendes, 2000). Motivated performance contexts have been studied extensively in the health and social psychology literature using the biopsychosocial (BPS) model of challenge and threat (Blascovich and Tomaka, 1996; Blascovich, 2008a), which articulates the psychological and physiological mechanisms by which stress can sometimes lead to more optimal performance and decision-making (associated with greater challenge orientation) and sometimes lead to poorer performance and decision-making (associated with greater threat orientation; for a review, see Hase et al., 2019). Consistent with this theorizing, existing empirical data on police officers shows that greater physiological arousal in stressful situations is sometimes associated with improvements in performance and decision-making (e.g., Verhage et al., 2018; Arble et al., 2019) and sometimes with decrements (e.g., Andersen and Gustafsberg, 2016). The BPS model may offer novel insights into the mechanisms by which police performance and decision-making can be optimized even in the face of unavoidable physiological arousal or stress.

This article briefly introduces the BPS model of challenge and threat and reviews recent empirical work that may shed light on the different ways that stress can influence police performance [broadly defined as the ability to handle critical incidents, involving situational awareness, verbal/non-verbal communication, self-control, or control of the public, (e.g., Brimmell et al., 2018; Arble et al., 2019)] and decision-making [defined as critical judgments to engage or not engage in a target behavior, (e.g., Correll et al., 2007a; Verhage et al., 2018)] across varying contexts, for both better and worse. We conclude by describing a research agenda to enhance the translational value of BPS research to better understand, and ultimately intervene, to improve, complex performance and decision-making behavior in the real world.

## The Biopsychosocial Model of Challenge and Threat

According to the BPS model, challenge and threat represent motivational orientations involving the interplay of affective and cognitive processes that result from the evaluation of situational and task demands relative to one’s available resources to cope with them (Blascovich and Mendes, 2000, 2010). More challenge-like states are experienced when a person evaluates his or her coping resources as meeting or exceeding the demands of the situation or task, while more threat-like states are experienced when a person evaluates the situation or task demands as exceeding his or her coping resources (Tomaka et al., 1993, 1997; Blascovich and Mendes, 2000, 2010). These appraisals (sometimes called evaluations) occur on a more subconscious and automatic level, are not under conscious control, and change dynamically over time as perceived demands or resources shift (see, Quigley et al., 2002; Seery, 2013). Originally, the factors contributing to the evaluation of task demands (e.g., perceptions of danger, uncertainty) were hypothesized as independent from those governing the evaluation of coping resources (e.g., dispositional factors, social support). However, more recent theorizing suggests that several factors are likely implicated in evaluations of both demands and resources (e.g., required effort, knowledge and skills, safety/danger; see Blascovich, 2008b). This is consistent with the idea that challenge and threat are not fixed and dichotomous, but rather malleable states that exist as opposing endpoints of a continuum (Seery, 2013; Jamieson et al., 2016).

The BPS model’s focus on appraisals of demands and resources developed out of Lazarus’ theory of cognitive appraisal (see Lazarus and Folkman, 1984; Folkman and Lazarus, 1985; Lazarus, 1991), which has been utilized in the policing literature to explore the role of cognitive appraisals in stress and performance among law enforcement personnel (e.g., Larsson et al., 1988; Harris et al., 2017). Lazarus introduced the terms “challenge” and “threat” as part of his theory, emphasizing that stress was not a unitary construct, but a system of responses that could be altered by changing one’s perception of a stressor (Lazarus and Folkman, 1984; Folkman and Lazarus, 1985). In Lazarus’ theory, “challenge” and “threat” reflect valenced appraisals that contributed to perceptions of a situation’s self-relevance and the potential for a situation to confer gains or losses, respectively (for review, see Jamieson et al., 2016). In the BPS model, challenge and threat responses *only* occur in self-relevant contexts and are associated with evaluations of the relative balance of demands and coping resources (Blascovich and Mendes, 2000, 2010). Critically, in the BPS model, unlike in Lazarus’ theory, the patterns of cognitive appraisals associated with challenge and threat are posited to engender reliably distinct patterns of physiological arousal (e.g., Blascovich and Tomaka, 1996; Blascovich and Mendes, 2000).

The basis for using physiological responses as indicators of challenge and threat in the BPS model is derived from the work of Dienstbier (1989) who theorized that quickly mobilizing energy resources during motivated performance, *via* the sympathetic-adrenomedullary axis, or SAM (as opposed to slowly *via* hypothalamic pituitary axis, or HPA), was a marker of “physiological toughness” because it was associated with favorable outcomes like increased performance, more emotional stability, and lower anxiety, leaving individuals more likely to appraise situations positively (for review see, Seery, 2011). Both challenge and threat states are theorized to involve activation of the SAM axis, and thus are associated with increases in indices of cardiovascular arousal (e.g., increased heart rate) and sympathetic nervous system control of the heart (i.e., ventricular contractility, measured as the inverse of pre-ejection period, the time between the electrical stimulus initiating ventricular contraction and opening of the aortic valve). Increases in ventricular contractility and heart rate are frequently interpreted as indicators of task engagement in motivated performance contexts (Seery, 2011, 2013). Experiencing a more challenge-like orientation is also associated with increased cardiac output (CO; volume of blood circulated per minute) accompanied by decreased systemic vascular resistance, typically measured as total peripheral resistance (TPR), the extent of overall constriction in the peripheral vasculature (Dienstbier, 1989; Blascovich and Mendes, 2000). In more challenge-like states, the heart beats harder and faster and moves blood more efficiently to the periphery, benefiting organ function and motor activity. Threat-like states involve activation of the SAM axis and the HPA, which inhibits decreases in vasoconstriction in the periphery. Thus, experiencing a more threat-like orientation is associated with no change or even increases in vascular resistance combined with more modest increases in cardiac output and ventricular contractility (i.e., blood flow is unable to reach and circulate in the peripheral vasculature as efficiently). While challenge-like patterns of physiological responding are interpreted as beneficial for the body, threat-like patterns of physiological responding are considered detrimental to energy mobilization (Tomaka et al., 1993, 1997). Moreover, the pattern of cardiovascular reactivity associated with threat-like states is theorized to have deleterious impacts on long-term cardiovascular health if experienced repeatedly over time (Mendes et al., 2007a,b; Blascovich, 2008a; Major et al., 2013).

## Research Using the Biopsychosocial Model

Although the BPS model has yet to be directly explored in real-world police decision-making contexts, challenge and threat orientations have been shown to influence decision-making and performance in other stressful, high-stakes environments. For example, challenge orientation (physiological and psychological) has been associated with better performance in situations that unfold over longer durations of time such as surgery (Moore et al., 2014), cricket and baseball seasons (Blascovich et al., 2004; Turner et al., 2013, respectively), flight simulation (Vine et al., 2015), negotiations (O’Connor et al., 2010), and semester grades (e.g., Seery et al., 2010). However, greater challenge orientation also confers benefits in visual attention (e.g., Moore et al., 2012; Vine et al., 2016), motor performance (e.g., Moore et al., 2012, 2013, 2014, 2015), attentional control (e.g., Vine et al., 2013, 2015, 2016), and working memory (e.g., Kelsey et al., 1999; Elzinga and Roelofs, 2005; Feinberg and Aiello, 2010). As such, the BPS model may offer unique insight for improving police performance under stress both in situations that unfold over longer durations of time (e.g., assessing danger during a robbery; Arble et al., 2019) and in rapid decision-making contexts (e.g., lethal force decisions; Correll et al., 2007a).

Although a complete review of the BPS literature examining performance under stress is outside the scope of this article, we briefly highlight empirical findings in content areas that are particularly relevant to police decision-making and performance given the current sociopolitical context in the United States.

### Stigmatization and Minority Status

Empirical studies with lay people have consistently demonstrated that, relative to White targets, participants are significantly quicker to shoot armed Black targets, significantly slower to “not shoot” unarmed Black targets, and have a lower shooting threshold for Black targets (i.e., tend to favor the “shoot” response) in computer-based shooting simulations (Correll et al., 2007a,b, 2011; Plant et al., 2011; Mekawi and Bresin, 2015). Laboratory research with real police officers on comparable tasks has demonstrated similar biases in reaction times for Black targets, though police officers generally demonstrate less bias in behavior and make fewer errors than lay individuals overall (see Correll et al., 2007a). Critically, research suggests these biases may be mitigated by a number of contextual and personal factors (for discussion, see Jetelina et al., 2017). For instance, police officers are less likely to demonstrate biases on shooting simulation tasks when they report having more positive interactions with Black people in their daily lives (Peruche and Plant, 2006) or report less overestimation of crime rates for minorities (Sadler et al., 2012), and studies with lay individuals have demonstrated reduced biases on tasks where counter-stereotypical targets (i.e., unarmed Black targets) are encountered more frequently (Correll et al., 2007b).

These findings on shooting simulation tasks are consistent with empirical work from the BPS model on challenge and threat responding during interactions with individuals from stigmatized groups. For example, Blascovich et al.’s (2001) “stigma-threat hypothesis,” posited that effort exerted during interactions with individuals from stigmatized groups increases because non-stigmatized individuals monitor their behavior more carefully to appear unaffected and avoid accusations of prejudice (Blascovich et al., 2001; Derks et al., 2011). Even for individuals who do not hold blatant prejudices, interactions with individuals from stigmatized groups can evoke knowledge of negative stereotypes, resulting in increasing efforts to monitor behavior and suppress stereotype-consistent thoughts (Devine, 1989; Wyer et al., 2000). Indeed, researchers have consistently demonstrated that participants show patterns of cognitive appraisal and cardiovascular activity consistent with more threatened orientations during interactions with individuals from stigmatized groups (Blascovich et al., 2001; Mendes et al., 2002, 2007a). For instance, White participants performed worse on a joint word-finding task and were more likely to produce cardiovascular patterns associated with threat-like states (e.g., increased TPR) when interacting with Black confederates or confederates of low socioeconomic status compared to White confederates (Mendes et al., 2002).

However, researchers have found that increased interaction with stigmatized groups is positively related to challenge-like physiological responses during intergroup interactions (Blascovich et al., 2001). These findings parallel those from studies involving police officers (Peruche and Plant, 2006) and lay individuals (Correll et al., 2007a), where increased exposures to individuals from stigmatized groups, in safe contexts, reduced racial bias in shooting simulation tasks. This suggests that interventions targeted toward increasing positive social interactions with individuals from stigmatized or minority populations may help increase the likelihood of exhibiting more challenge-like orientations during intergroup interactions to the benefit of performance and decision-making.

### Power

Research on the BPS model has examined the nuanced ways in which social status and power influence patterns of biopsychological activity. Specifically, individuals high in social status or power tend to experience more challenge-like states during social interactions with people of lower status (e.g., Scheepers and Ellemers, 2005; Scheepers, 2009; Scheepers et al., 2012). For example, participants prompted to recall incidents in their lives where they had a lot of power or who were randomly assigned to a high-power role (e.g., given more leverage in a negotiation task) exhibited more challenge-like appraisals and cardiovascular activity than participants randomly assigned to low-power comparison conditions (Scheepers et al., 2012). Consistently, Akinola and Mendes (2012) found that police officers who self-reported higher social status exhibited more approach-oriented or challenge-like patterns of physiological reactivity, including increased heart rate, cardiac output, and testosterone reactivity, during a simulated interaction with a disgruntled citizen.

Critically, research suggests that the relationship between power and biopsychological responding relies on the stability of the power hierarchy; those high in status show more threat-like responses when their status is perceived as unstable or illegitimate (Scheepers and Ellemers, 2005; Scheepers, 2009). These findings may offer insight into why tense situations can escalate quickly depending on social dynamics. Specifically, when police officers feel secure in their status as an authority figure and do not believe they are being undermined by a suspect or civilian, they may be more likely to engender challenge-like orientations to the benefit of decision-making and performance in that context. Moreover, research suggests that, when individuals’ social identities are threatened, engaging in self-affirmation strategies engenders more challenge-like patterns of physiological reactivity (Derks et al., 2011). Thus, it may prove beneficial to develop interventions to help police officers maintain stable perceptions of their status (e.g., by engaging in self-affirmation strategies) even when interacting with suspects or other civilians who are questioning their legitimacy or status.

## The Biopsychosocial Model and Police Decision-Making: A Research Agenda

**Figure 1:**
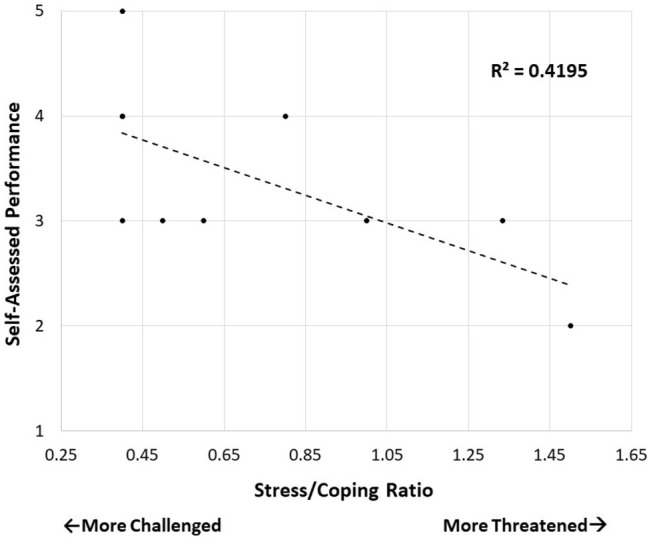
Stress/coping ratios predict self-assessed performance (rated on a 5-point scale) during an emergency training drill. Ratio of self-reported stress to self-reported coping ability is shown on the *x*-axis, with higher numbers indicating more threat-like appraisals and lower numbers indicating more challenge-like appraisals. An estimate of *R*^2^ is provided in figure, though should be interpreted with caution due to sample size.

Although the BPS model has proven useful in examining performance under stress across a variety of motivated performance contexts (see Behnke and Kaczmarek, 2018; Hase et al., 2019), there remains a critical need for translational research investigating its utility in real-world situations, specifically in the context of police interactions and decision-making. A recent field study demonstrated the viability of using the BPS model to examine and predict biopsychological responding among first responders, including police officers, in an ecologically valid, high-stress situation: a multi-faceted drill simulating the response to a plane making an emergency landing with a fire and injured passengers onboard (Wormwood, 2019). Consistent with the BPS model, more challenge-like appraisals among first responders were associated with better self-assessed performance during the drill (Figure 1).

Data from this field study highlight several critical theoretical and methodological considerations for researchers interested in pursuing translational research examining the relevance of the BPS model in realistic decision-making and performance scenarios. First, the vast majority of research using the BPS model has focused on group comparisons between samples of individuals exhibiting more threatened versus more challenged appraisals, failing to examine challenge and threat responses at the level of the individual (though see Quigley et al., 2002). As a result, little is known about why, given identical circumstances, one individual may be more challenged while another is more threatened (for review, see Kilby et al., 2018). Note the presence of robust individual differences observed in the recent field study, even within a highly evocative, personally-relevant context (Figure 1; Wormwood, 2019). According to Dienstbier (1989), exposure to repeated intermittent stressors could result in a proneness to challenge-like responding under stress, a pattern of response linked to both increased emotional stability and immune system enhancement (Seery, 2013). This is consistent with a number of clinical and therapeutic approaches (e.g., cognitive behavior therapy, desensitization therapy, stress inoculation training) which posit that trainings involving repeated exposure to small stressors that the individual can cope with successfully may bolster more effective, challenge-like performance in the face of future, unknown stressors.

**Figure 2:**
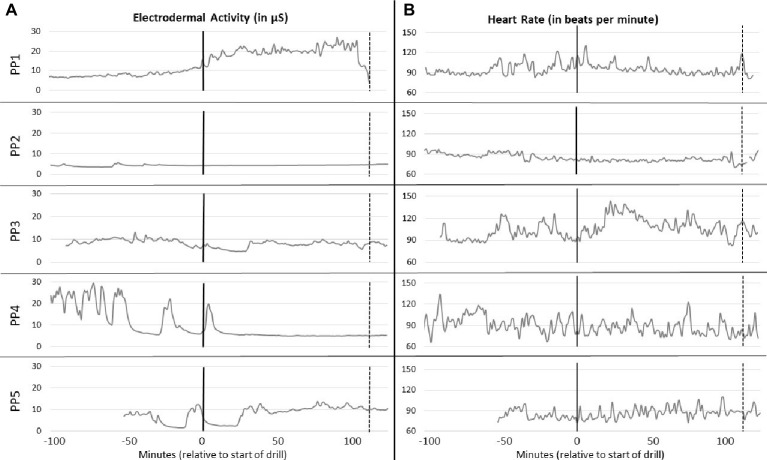
Electrodermal activity and estimated heart rate for five individuals throughout emergency training drill. Time in minutes relative to the start of the drill is shown on the *x*-axis. The start of the drill is marked with a solid vertical line and the end of the drill is marked with a dashed vertical line on each graph. **(A)** shows electrodermal activity in microsiemens (μS) for the five participants (PP1, PP2, PP3, PP4, and PP5) throughout the study. **(B)** shows estimated heart rate (HR) in beats per minute for the same five participants throughout the study. Each row contains EDA and HR data for the same participant. For both physiological variables, an average value was taken for each 1 min of recording, and these averages are plotted here.

In addition, research using the BPS model has focused almost exclusively on static assessments of challenge and threat responding and has not examined how patterns of physiological activity or cognitive appraisals change dynamically over time as a stressful context unfolds. The recent field study revealed dynamic changes in physiological and SNS arousal over time throughout the training, and these patterns of change varied markedly across individuals (Figure 2; Wormwood, 2019). The focus on static assessment means important information about early detection and genesis of challenge-like and threat-like states in the face of stressors remain unknown. Information concerning the time course of psychobiological states might be useful for designing early intervention systems involving biofeedback or for identifying critical time points at which individual interventions might be most effective at mitigating threat-like physiological patterns as they unfold in real time.

Future translational work would also benefit from the inclusion of complementary methodological approaches used less frequently in research on the BPS model. For instance, audio-visual data recorded continuously during a motivated performance context would be invaluable for elucidating how and why challenge and threat orientations shift dynamically as a context unfolds. Qualitative interviews with participants could shed light on important individual differences related to the tendency to experience more challenge-like orientations under stress, or could suggest potential mechanisms for future exploration of factors contributing to appraisals of demands and/or resources. Considering the myriad contexts in which police officers must optimize performance and decision-making, future translational research would benefit from the inclusion of more diverse, real-world contexts (e.g., situations involving different combinations of cognitive, affective, social, and motoric features). Comparing diverse scenarios may offer insights on the psychological and physiological mechanisms by which challenge and threat orientations influence performance or decision-making across contexts.

## Conclusion

The BPS model appears well-suited for studying the psychophysiology of police performance and decision-making because challenge- and threat-like states are relevant across a wide range of social evaluative and motivated performance domains (e.g., Behnke and Kaczmarek, 2018), are associated with consistent patterns of cognitive appraisal and cardiovascular reactivity (Blascovich, 2008a), and have been shown to predict important behavioral and decision-making outcomes in stressful contexts (see Hase et al., 2019). Moreover, while threat-like states have not been *directly* linked to health outcomes, prolonged physiological activation (particularly in the HPA) can result in profound negative health consequences (e.g., McEwen, 1998). Thus, research examining challenge and threat responding among police officers in the line of duty stands to improve early detection of individuals at risk for the negative health outcomes (e.g., cardiovascular disease) that are associated with careers in law enforcement (Hartley et al., 2011).

## Author Contributions

DK and JW drafted the manuscript. ES provided substantial edits and feedback on the manuscript.

### Conflict of Interest Statement

The authors declare that the research was conducted in the absence of any commercial or financial relationships that could be construed as a potential conflict of interest.
